# Case of Common Continued (Typhoid) Fever

**Published:** 1849-02

**Authors:** Austin Flint


*Case of common continued (Typhoid) Fever. Autopsy.* By AUSTIN FLINT, M.D.—The following case of fever is interesting, as presenting, in an unusually prominent degree, active delirium, which persisted through the whole career of the disease. In this respect it strikingly resembled the last of the series of cases reported for the November No. of this Journal, in which an opportunity for autopsical examination could not be obtained. As we are able in this case, to present, in connection with the history, the appearances found after death, we have thought it worthy of being reported.

John Stellwagon, a German, aged 20 years, having recently come to this country, of good habits, a carriage maker, entered the hospital of the Sisters of Charity, Nov. 29, 1848. As he was wholly unacquainted with the English language, I was unable to obtain any account of his disease from his own lips. I learned from his friends, that he had been gradually becoming ill for a couple of weeks; that he had taken to his bed two days before his entrance; that he had been bled on the morning of the day he entered the hospital; that he had previously taken purgative medicine; and that he had manifested more or less aberration of mind for several days.

The first record of symptoms was made on the 30th. He was then (morning) tranquil; lying on back; countenance presenting nothing worthy of note; respiration normal; tongue coated and dry in the centre; skin warm and dry; pulse 120, well developed; abdomen not distended; no eruption visible; no tenderness on pressure; no gurgling, but the latter symptom had been observed the previous evening by the resident pupil; was not then thirsty, but had been so during the past night, asking very often for drink. No disposition to somnolency. Surface of body presented moderate hyperæmia, leaving whiteness on pressure, which slowly disappeared.

Treatment:—

Pulv. Doveri, grs. v.
Pulv. Aromat. grs. ii.

every six hours. Sponge the surface with lukewarm water, and allow cold water to be drank freely.

Dec. 1st. Says he is better. Expression of countenance wild. Was actively delirious through the night, as he had been during the preceding night, getting out of bed, and, if not closely guarded, attempting to get out of the window. Endeavors this morning frequently to get out of bed. Tongue much reddened; papillary eminences elevated, especially in the centre; somewhat dry; slightly furred. Pulse 136, less developed than yesterday. Two dejections occurred in bed during the night. Abdomen slightly meteorized; no tenderness on pressure. Four or five rose spots counted on abdomen and chest. Some redness of cheeks. Eyes not injected; pupils unnaturally dilated; no evidence of undue sensitiveness to light. Temperature of head not relatively elevated.

℞. S. Morphiæ, gr. $\frac{1}{4}$,

P. Camphoræ, grs. iv,

P. Sacch. Alb., grs. v.

every four or six hours, according to wakefulness or delirium.

He frequently attempted to get out of bed during my visit. He does not appear to understand the import of questions addressed to him, replying incoherently. Protrudes his tongue when requested.

Evening. Delirium continues; getting out of bed unless constanly watched. On one occasion, when attention was diverted from him for a few moments, he was found to have crept beneath the bed, apparently from terror. Talks incoherently; pulse very frequent; skin hot and dry; tongue dry; no sensitiveness to light; complains of no pain, and declares that he is perfectly well.

Treatment;—Emp. vesicat. to Nuchæ 6x3. Repeat morpia and camphor every three hours, if vigilance and active delirium continue. Cold applications to head, renewing very frequently. Thin milk porridge for diet.

2d. Active delirium continued through the night. No dejection; passed urine freely; mutters; Carphologia; copious perspiration; tongue moist; protrudes it when requested; pulse 160, small. Abdomen moderately tympanitic, no more rose spots visible. Takes drink readily. Respirations not accelerated.

Treatment continued, with addition of Madeira wine ℥ss hourly.

Evening. Perspiring copiously; skin warm; pulse so frequent as to be scarcely enumerated; mutters incoherently. Occasionally sleeps for a few moments, and heavily; countenance sunken; protrudes his tongue readily, which is moist; some sordes on the teeth; respirations 30; abdomen not distended.

Carb. ammon. grs. v.

Aquæ Puræ ℥ss, every two hours.

Brandy ℥ss, every two hours.

Increase both brandy and ammonia if he continues to sink.

Died at 11 o'clock, P.M.

*Autopsy twelve hours after Death. Head.*—Moderate adhesion of dura mater. Arachnoid membrane diaphanous; a little serum beneath the membrane at dependent portion of brain. Some effusion at base of brain, quantity could not be estimated, being commingled with blood which escaped on removing the brain from the skull. Large veins between convolutions, congested. A considerable number of red points on section of cerebral substance. Ventricles empty. Consistence of brain's substance normal.

*Chest.*—Slight, old adhesions over small space in right chest. Several ounces of sanguinolent effusion (say 5 or 6) in left chest. No adhesion in this chest; no lymph. Pleural membrane presented nothing to attract notice. Lungs on both sides free from morbid appearances, presenting the amount of hypostatic congestion generally found in autopsical examinations; crepitating throughout.

*Heart.*—Slight effusion of transparent serum. Organ rather below normal size. Left ventricle empty. Left auricle contained a small quantity of fluid blood. Right ventricle contained fluid blood, with some small, soft coagula. Right auricle moderately distended with blood, mostly fluid.

*Abdomen.*—Colon and cœcum greatly distended with gas; small intestines moderately so. Dividing the ileum close to the cœcum, and, lengthwise, for several feet upward, the glands of Peyer were found to be enlarged, projecting two or three lines above the surface of the surrounding mucous membrane. From fifteen to twenty patches were counted, the enlargement diminishing, progressively, upward. No ulcerations, nor discoloration, perceptible. Mucous surface appeared healthy. Numerous enlarged solitary glands visible, some as large as small peas. Mesenteric glands, in portions of mesentery corresponding to diseased glands of Peyer, greatly enlarged. At lower portion of ileum they were as large as filberts; their enlargement was less and less, in correspondence with diminution of disease of Peyer's glands upward, and they ceased to be visible at a point corresponding to the limit of the latter. On section of the Mesenteric glands, some presented red, and some white color; none were found to contain pus or other fluid.

*Stomach.*—Presented punctated redness, or ecchymoses. No capilliform redness. Size normal. Mucous membrane softened. Several ulcerations varying in size and form; the largest half an inch in length, and three or four lines in width, superficial. apparently, having penetrated only the mucous coat. These appearances were limited to the larger curvature. The organ at this part was easily torn, a rent occurring in removing it from its vascular splenic attachments.

*Spleen* somewhat enlarged, but not softened.

*Liver* congested. Otherwise normal.

*Remarks.*—The history of the foregoing case shows a predominance and persistenee of active delirious excitement, which is not common in cases of continued fever in this region. The intestinal lesions furnished a fine exemplification of those which are regarded by Louis, and others, as constituting the "*anatomical characteristic*" of typhoid fever, as distinguished from typhus. The non-softening of the spleen was exceptional to the general rule. The pathological condition of the stomach is to be regarded as an accidental element of the case. The chief point of interest relates to the absence of evidences of inflammation in the encephalic structures, considered in connection with the cerebral symptoms during life.—*Buffalo Medical Journal.*

